# Emetic *Bacillus cereus* Are More Volatile Than Thought: Recent Foodborne Outbreaks and Prevalence Studies in Bavaria (2007–2013)

**DOI:** 10.1155/2014/465603

**Published:** 2014-05-08

**Authors:** Ute Messelhäusser, Elrike Frenzel, Claudia Blöchinger, Renate Zucker, Peter Kämpf, Monika Ehling-Schulz

**Affiliations:** ^1^Bavarian Health and Food Safety Authority, Veterinärstr. 2, 85764 Oberschleißheim, Germany; ^2^Functional Microbiology, IBMH, Department of Pathobiology, University of Veterinary Medicine, Veterinärplatz 1, 1210 Vienna, Austria; ^3^Microbiology Unit, Center for Nutrition and Food Research ZIEL, Technical University of Munich, 85350 Freising, Germany

## Abstract

Several *Bacillus cereus* strains possess the genetic fittings to produce two different types of toxins, the heat-stable cereulide or different heat-labile proteins with enterotoxigenic potential. Unlike the diarrheal toxins, cereulide is (pre-)formed in food and can cause foodborne intoxications shortly after ingestion of contaminated food. Based on the widely self-limiting character of cereulide intoxications and rarely performed differential diagnostic in routine laboratories, the real incidence is largely unknown. Therefore, during a 7-year period about 4.300 food samples linked to foodborne illness with a preliminary report of vomiting as well as food analysed in the context of monitoring programs were investigated to determine the prevalence of emetic *B. cereus* in food environments. In addition, a *lux*-based real-time monitoring system was employed to assess the significance of the detection of emetic strains in different food matrices and to determine the actual risk of cereulide toxin production in different types of food. This comprehensive study showed that emetic strains are much more volatile than previously thought. Our survey highlights the importance and need of novel strategies to move from the currently taxonomic-driven diagnostic to more risk orientated diagnostics to improve food and consumer safety.

## 1. Introduction

Cereulide, an emesis-inducing toxin produced by a fairly homogenous group of* B. cereus* strains called “emetic* B. cereus*,” is a small heat-stable cyclic peptide [1]. The emetic poisoning caused by cereulide is usually characterized by vomiting starting after 0.5 hour to six hours after consumption of the contaminated food. Intoxications proceed mostly with mild symptoms and last normally not more than one day, but severe cases requiring hospitalization are increasingly reported (for review see Ehling-Schulz et al., 2004 [2], and Ehling-Schulz et al., 2011 [3]).

Because of the short period of illness, the emetic syndrome caused by* B. cereus* is presumably underreported [4]. In addition, the symptoms of an emetic intoxication caused by* B. cereus* parallel the symptoms caused by* S. aureus* enterotoxins, bearing the risk of misdiagnosis of the disease. In the year 2011 the European Food Safety Authority (EFSA) reported an increase of 122.2% in the number of foodborne intoxications and toxicoinfections caused by* B. cereus* in Europe. The overall reporting rate was 0.04 cases per 100.000 inhabitants [5]. Even if intoxication with the emetic toxin cereulide in most cases produces only mild symptoms, consistently also fatal cases are reported [6–8].

Although* B. cereus* is an ubiquitous spore former, emetic strains are rarely found in the environment and their natural niches and entrance points into the food production and processing are largely unknown [4]. So far, mostly high-carb food matrices, such as rice and pasta, as well as milk and dairy products, have been investigated for the presence of emetic strains of* B. cereus* [9–13], whereas other food matrices have rarely been included in the analyses. To improve HACCP-based concepts and prevent foodborne intoxications caused by emetic* B. cereus*, information on the general prevalence of emetic strains in foods of different origin is of utmost importance and data on the risk of toxin formation in different food categories are required.

This study therefore aimed to (i) investigate the prevalence of emetic* B. cereus* strains in a wide range of food matrices, covering foods from plant as well as animal origin, to identify potential contamination sources, and to (ii) facilitate hazard identification by exploring the potential of diverse food matrices for the risk of cereulide toxin production. In this context, a perennial survey from 2007 to 2013 was carried out, including food samples connected to emesis-related foodborne illnesses as well as samples not related to foodborne outbreaks. By using an* in situ* bioassay indicative of cereulide production levels, a general scheme for categorizing foods with respect to their risk of cereulide production was generated.

## 2. Material and Methods

### 2.1. Sample Material

Between the years 2007 and 2013 3.564 food samples from Bavaria were analysed for the presence of emetic* B. cereus* strains in the context of foodborne illness or outbreaks where the consumers showed symptoms of vomiting. The majority of samples were taken from the household of the diseased consumers and from restaurants, canteens, and catering companies. Additionally, the presence of emetic* B. cereus* strains in different food matrices (*n* = 742) was investigated in the scope of different monitoring programs. Food categories for the monitoring were chosen from both food of animal origin and food of plant origin. All samples were examined before their expiry date.

### 2.2. Microbiological Detection of* B. cereus *and Identification of the Cereulide Synthetase Gene* ces*

Emetic* B. cereus* strains were detected with qualitative and quantitative methods (for details see Ehling-Schulz et al., 2011 [3]). The qualitative detection was done weighting 10 g of sample material into 90 mL of tryptone-peptone-glucose-yeast (TPGY) broth and incubating at 30°C under aerobic conditions. After 24 h of cultivation 1 mL of the enrichment broth was taken for the molecular detection of the* ces* genes, which encode the nonribosomal synthetase responsible for the production of the peptide toxin cereulide. For detection of* ces*, a previously described probe-based diagnostic real-time-PCR assay was used [9, 15].

The quantitative detection of presumptive* B. cereus* was carried out using standard reference culture methods recommended by the International Organisation of Standardization (ISO) and the U.S. Food and Drug Administration (FDA). Samples were investigated using spiral plate count method on the Mossel agar [16, 17] or with a 3-tube 3-dilution most probable number (MPN) method [18–21]. Presumptive* B. cereus* colonies were further differentiated by the detection of the* ces* gene either by real-time-PCR as described above or by using a conventional PCR system according to Ehling-Schulz et al., 2004 [22]. Depending on the results of these reactions the number of colony-forming units per gram (cfu/g) or MPN of emetic* B. cereus* cells per gram of sample was calculated following the standard methods recommended by FDA and ISO [16, 17, 21].

### 2.3. Bioassay-Based Risk Categorization of Foods

Analysis of the potential of food matrices to support cereulide production was performed by artificial contamination of 30 g portions with the bioluminescent* B. cereus lux *reporter strain F4810/72(pMDX[P_1_/*luxABCDE*]) and an IVIS camera system as described earlier [23, 24]. Foods were provided by diverse manufacturers or were obtained from local consumer markets. In the case of powders and freeze-dried products (e.g., infant formulas and instant potato powder) or raw materials (e.g., rice and pasta) foods were prepared according to the manufacturers' instructions thereby simulating common household conditions. The contents of preportioned packaging units (e.g., single-sliced cheese or biscuit snacks) were combined and blended for 3 min with a stomacher to obtain homogenous testing matrices. Dry foods, such as dates, apricots, cocoa powder, and herbal salt, were additionally soaked with sterile water or pasteurized milk (1.5% fat content) as indicated. Matrices were filled into Petri dishes and inoculated to a final reporter strain cell count of 10^3^ CFU per gram. After an incubation step for 24 hours at 24°C, the luciferase signal intensities were quantified with a photon-counting intensified-charge-coupled-device (ICCD) camera (model 2400-32; Hamamatsu Photonics) and are shown as false-color renderings that were superimposed on gray-scale images of the respective food sample.

## 3. Results and Discussion 

This study was designed to get a comprehensive overview of the prevalence of emetic* B. cereus* strains in both food samples from supposed foodborne intoxications and food samples from general food monitoring programs. These data should provide a profound basis for a better risk assessment concerning the emetic syndrome caused by cereulide producing emetic* B. cereus* strains. In addition, the influence of food matrix properties on cereulide production was evaluated using a previously established* lux* reporter system [24].

### 3.1. Prevalence of Emetic* B. cereus* in Foods Linked to FoodBorne Intoxications and in Nonfood Intoxication Associated Food Samples

**Figure 1:**
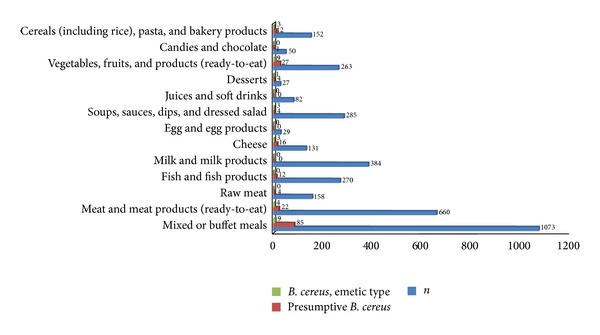
Presumptive* Bacillus cereus* and emetic strains in different food matrices investigated in the context of supposed foodborne intoxications.

Because most studies hitherto targeted only a very limited range of food matrices, such as rice and pasta (e.g., [25–27]), and samples were collected from very specific sites or during very short sampling periods (see e.g., [13]), prevalence data covering samples from different years and diverse food matrices are still missing. However, in the context of preventive consumer protection policy and for a comprehensive risk assessment, data about the prevalence of emetic strains in different food categories from a perennial sampling period are required. We therefore analysed 3.654 food samples obtained from suspected foodborne illness with a preliminary report of vomiting shortly (within a period from thirty minutes to six hours) after consumption of the suspected meal over a period of 7 years (2007 to 2013). The analysed samples covered a broad variety of food categories (Figure 1). Presumptive* B. cereus* was detected in 187 samples (5%) and emetic* B. cereus* strains were detected in 32 samples (1%). Interestingly, emetic strains were not only detected in farinaceous foods commonly linked to cereulide intoxication (e.g., [1, 6, 8]) but also in vegetables, fruit products, sauces, soups, and salads as well as in cheese and meat products (Figure 1). Recently, Doménech-Sánchez et al. [28] reported on an emetic outbreak linked to the consumption of tuna fish. These results emphasize that more data on the prevalence of emetic* B. cereus* in different types of foods are needed to decipher potential contamination sources.

In most samples tested positive for* B. cereus, *which have been analysed in the context of foodborne intoxications, emetic strains were found in levels ≤10^2^ cfu/g food matrix (see Table 1). All of these samples were tested negative for the presence of other foodborne pathogens, including* S. aureus* and its enterotoxins (data not shown). It is therefore assumed that* B. cereus* was indeed the etiological agent of the reported outbreaks. The detection of emetic* B. cereus* in low levels in samples from suspected foodborne illness could be an indication that the bacteria themselves were reduced by the food production and processing procedure, but the preformed heat- and acid-stable toxin cereulide was not eliminated or inactivated. In addition, it is known that the capability of toxin formation varies significantly among emetic* B. cereus* strains and the actual toxin production depends on external parameters [11, 23, 29, 30]. These examples highlight the need of novel diagnostic strategies, moving from taxonomy to more risk orientated differential diagnostics (for review see Ehling-Schulz and Messelhäusser, 2013 [31]).

**Figure 2:**
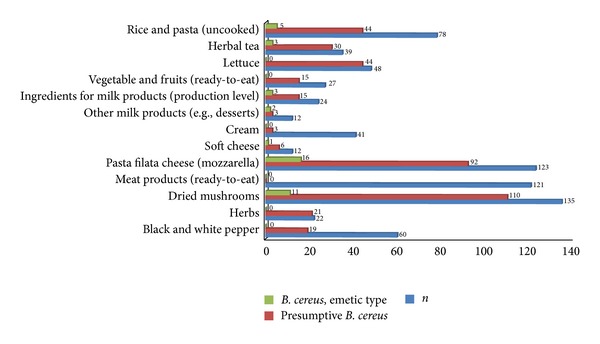
Presumptive and emetic* B. cereus* in different food matrices investigated in the context of monitoring programs.

To gain a deeper insight into food associated natural niches of emetic* B. cereus* and potential contamination sources, 742 food samples of animal and plant origin were investigated for the presence of emetic* B. cereus* strains within different monitoring programs (Figure 2). For food of animal origin, samples were grouped in categories that have been reported in the context of foodborne illness, for example, ready-to-eat meat products, cheese, and cream. For food of plant origin, food matrices were investigated that could be possible contamination sources for ready-to-eat food, such as herbs, spices, and dried mushrooms or fresh foods, such as lettuce, fruits, and vegetables. Emetic strains were most frequently found in pasta filata cheese obtained from retail level (13%), in dried mushrooms (8%), and in herbal teas (8%). The detection rates in these matrices were even higher than in uncooked rice and pasta (6%), whereof also 78 samples were investigated. Overall, 10% of presumptive* B. cereus* strains, isolated in the context of monitoring programs, possess the* ces* gene and therefore the ability to produce cereulide toxin. These prevalence rates are slightly higher than the ones reported from previous studies (e.g., [25, 27]). One explanation might be that emetic* B. cereus* strains are easily overlooked in routine diagnostic since they frequently show an atypical phenotype and might, in addition, be outcompeted on nonselective agar media often used in microbial diagnostics [32]. The food category investigated could also significantly influence the percentage of emetic isolates detected. For instance, as our study showed (in food categories for which more than 50 samples were investigated) the percentage of emetic strains isolated from different food matrices varied between 10% (dried mushrooms) and 17% (pasta filata cheese) (see Figure 2).

However, not only the presence of strains but also the potential of food matrices to support cereulide synthesis should be considered for an accurate risk assessment, since unavoidable low-level contaminations with the spore formers might lead to intoxications or even large-scale outbreaks in cases of improper storage and handling of prepared meals. Previous work showed that the risk of cereulide production is strongly connected with external parameters and varies significantly among different types of model foods that have been investigated so far [23, 24, 30].

### 3.2. Broad-Scale Risk Categorization of Food Matrices concerning Cereulide Synthesis

Although the EFSA stressed the necessity of identifying categories of foods that may pose a risk for human health with respect to cereulide contamination [4], a comprehensive evaluation of food matrices was hampered due to laborious, time-consuming, and error-prone methods to quantify cereulide amounts in foodstuffs. Recently, a SIDA-based method allowing the quantitative detection of cereulide has been developed [33]. However, alternative high-throughput methods to estimate the risk of toxin production in diverse food matrices are needed. The* lux*-based reporter system for real-time monitoring of toxin gene expression described by Dommel et al. [24] might represent an interesting tool in the latter context. We previously showed that cereulide production in model food matrices is proportional to the intensity of the bioluminescence signals emitted by the engineered* B. cereus* reporter strain [23, 24].

In this study, we employed the* lux* reporter system for the analysis of a total of 70 retail products in order to decipher abiotic and nutritional factors, either promoting or suppressing toxin synthesis. Luciferase signals were quantified with a software-assisted region-of-interest (ROI) analysis and foods were categorized into three main classes regarding their toxin formation capability: high-risk, risk, and low-risk foods (Table 2, Figures S1–S3 in Supplementary Material available online at http://dx.doi.org/10.1155/2014/465603). Derived mean ROI values of each risk category and the corresponding determined threshold values are listed in Table S1. The bioassay revealed that 44% of the foods could be categorized as high-risk foods, while the remaining 20% and 36% were categorized as risk or low-risk foods, respectively (Table 2). Products classified as being insensitive were dairy based, displayed a low pH value (e.g., cream cheese and unsweetened quark), had a high fat content like chocolate and nut spread, and/or were characterized by low water availability or high osmolarity (e.g., dried fruits and 10% herbal salt solution). Earlier studies showed that growth of* B. cereus* was suppressed in foods with pH values below 5.0 [34–36], which is in line with our low-risk classification of matrices that had pH values around 4.3 to 4.8, such as the whey drinks. The combination of neutral pH values and medium a_w_-values with high amounts of fat and cocoa was found to be indicative of the group of products being at medium risk of toxin synthesis (Table 2 and Figure S2). Likewise, proteinaceous foodstuff containing high fat amounts, such as minced beef or milk powder-based processed cheeses, fell in the same category. This is in agreement with a previous study [37] showing that cereulide was produced in small quantities in artificially contaminated meat products. The same study also supports our results concerning the pasteurized milk: usually, only low to medium cereulide levels are produced under stationary conditions at room temperature [30, 37]. Additionally, dairy products dulcified with glucose or fructose (quark desserts, cream-filled soft biscuits) fell in the intermediate class in terms of the risk for cereulide production. It was shown previously that glucose had a stimulating effect on cereulide synthesis [4]. The group of high-risk products comprised farinaceous foods, as well as powdered products that were reconstituted with water or milk (Table 2, Figure S3). Dairy- and cereal-based infant food formulas, which were additionally enriched with vitamins or trace elements, promoted exceptional high* ces *promoter activities. The latter indicates that a combination of readily available saccharides, vitamins, and macronutrients in a pH neutral environment may stimulate toxin formation. Indeed, cereulide was detected in high levels in farinaceous matrices or systems containing high amounts of K^+^ ions and vitamins [10, 38].

**Figure 3:**
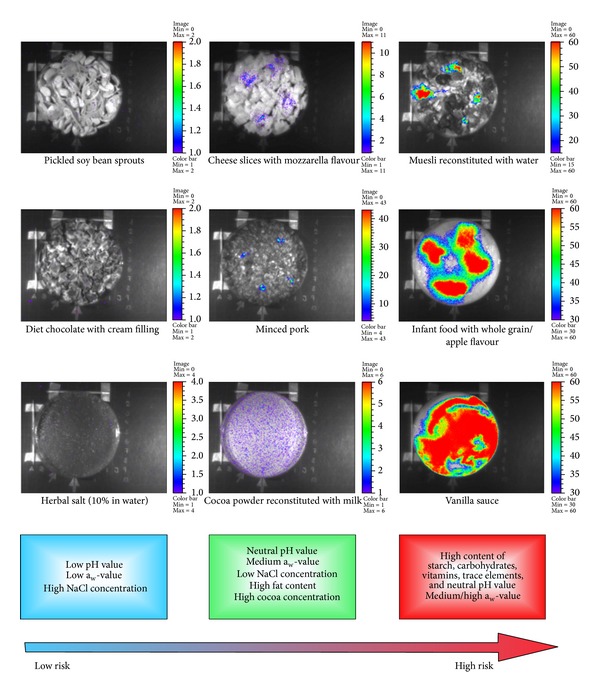
Scheme for abiotic factors influencing the activity of the* ces* NRPS promoter driving the synthesis of cereulide. The parameters were deduced from the examination of 70 foods and food ingredient using an emetic* lux* reporter strain [10]. The arrow denotes an increasing toxin formation capability with respect to the food composition. Examples of typical food matrices for each category are shown.

A summary of food characteristics commonly observed in the three categories is provided in Figure 3. This generalized scheme allows a basic preevaluation of foods and their ingredients concerning their capability to support cereulide formation and should facilitate hazard identification in terms of HACCP concepts.

## 4. Conclusion

Overall, our results indicate that emetic* B. cereus* strains occur more frequently and in a much broader diversity of foods than noticed so far. In addition, the* lux*-based real-time monitoring assay turned out to be a valuable tool for assessing the actual risk of cereulide toxin production in different types of food, allowing us to set up a general scheme for the categorizing of foods with respect to their cereulide production risk. Our survey of presumptive emetic* B. cereus* foodborne outbreaks also showed that the risk of an emetic syndrome caused by the* B. cereus* cereulide toxin is not restricted to high-carb foods, such as pasta and rice. Much more attention must be paid to other foods, especially the ones supporting cereulide production, as shown by the* lux *reporter assay.

## Supplementary Material

Figures S1, S2, S3: Examples of foods categorized being at low risk (Si), at risk (S2) or at high risk (S3) to promote cereulide formation in the presence of emetic *B. cereus* strains.
The bioassay-based risk categorization was performed by inoculating 70 retail products and food ingredients with a lux based B. cereus cereulide reporter strain. The luminescence intensity, which corresponds to the amount of synthesized cereulide [24], was quantified in situ after incubation of the samples for 24 hours at 24°C (for details, see Material and Methods). Food samples were grouped into three main classes regarding their toxin formation capability via a software-assisted region-of-interest (ROI) analysis. The bioassay revealed that 44% of the foods could be categorized as high-risk foods, while the remaining 20% and 36% were categorized as risk or low-risk foods, respectively. Derived mean ROI values of each risk category and the corresponding determined threshold values are listed in Table S1.

## Figures and Tables

**Table 1:** Examples for potentially foodborne diseases caused by emetic *B. cereus* in Bavaria between the years 2007 and 2013.

Year	Diseased persons	Place	Food matrix	Level of emetic *B. cereus* (cfu/g)
2007	Several students after a cooking lesson at school	School kitchen	Hard cheese	<100 (only positive using a qualitative detection method, but detection of 2 *μ*g cereulid/g*)

2007	One adult	Restaurant	Cooked pasta	3.8 × 10^5^

2008	Several students	School canteen	Paprika filled with meat and rice	<100 (only positive using a qualitative detection method)

2009	One adult	Household	Cooked potatoes	<100 (only positive using a qualitative detection method)

2010	One adult	Restaurant	Cooked pasta with oysters	<100 (only positive using a qualitative detection method)

2010	Several adults	Canteen	Poulard breast in tomato sauce	<100 (only positive using a qualitative detection method)

2010	Several adults	Catering	Chana masala (cooked chickpea) with baked potatoes in curry sauce and cooked rice	Cooked rice: 2.8 × 10^4^ (1 *μ*g cereulid/g*) Cooked chickpea: <10 (only positive using a qualitative detection method, but detection of 0.3 *μ*g cereulid/g*); see also Ehling-Schulz and Messelhaeusser, 2012 [14]

2011	Several children (1 to 3 years old)	Nursery school	Cooked pasta with tomato sauce	6.8 × 10^6^

2011	Two adults	Restaurants	Cooked pork meat with potatoes	1.0 × 10^2^

2011	One adult	Household	Cured and smoked meat	1.0 × 10^2^

2012	Several students	Canteen	Raspberry quark	1.4 × 10^2^

2012	One adult	Household	cooked mushrooms	1.9 × 10^7^

2013	Several adults	Catering at a wedding	Vitello tonnato	6.1 × 10^7^

*Currently, no officially validated method for the quantitative detection of cereulide in food matrices is available; therefore quantitative data on cereulide toxin are only shown for selected samples. However, recently a European initiative has been started to establish appropriate ISO methods (CEN/TC 275/WG 6).

**Table 2:** Bioassay-based categorization of 70 retail foods according to their potential for supporting cereulide production. Bioluminescence intensity produced by the cereulide synthesis reporter strain F4810/72(pMDX[P_1_/*luxABCDE*]) was measured after 24 hours of incubation at 24°C. Representative images are shown in Figure 3 and Figures S1–S3. Threshold values established for risk categorization are listed in Table S1.

Low-risk foods	Risk foods	High-risk foods
Dried apricots	Reconstituted milk powder (organic)	Cereal-based reconstituted infant food (fruit flavour)
Dried apricots rehydrated with water	Dried dates rehydrated with water	Cereal-based reconstituted infant food
Infant food with yoghurt and fruits	Cheese slices with suisse flavour	Cereal-based reconstituted infant food (whole grain/apple flavour)
Crème fraîche	Cheese slices with mozzarella flavour	Dessert creme with cream/coffee flavour
Crème fraîche with herbs	Minced pork	Dessert creme with caramel flavour
Dried dates	Minced veal	Diet drink with vanilla flavour
Diet chocolate with cream filling	Cocoa powder with milk	Muesli with water
Cottage cheese (whole fat content)	Herbal salt (1% in water)	Muesli with milk
Fresh cheese (natural)	Latte macchiato drink	Semolina pudding (natural)
Fresh cheese with herbs	Camembert cheese (60% fat content)	Semolina pudding (vanilla flavour)
Fresh cheese with chilli flavour	Chocolate mousse	Semolina pudding (cinnamon flavour)
Yoghurt of fresh cheese with fruits	Pasteurized milk (1.5% fat content)	Boiled Jasmin rice (organic grains)
Yoghurt of fresh cheese with vanilla/fruit	Pasteurized cream (30% fat content)	Boiled Jasmin rice (parboiled grains)
Yoghurt of fresh cheese with raspberry	Chocolate biscuit with milk cream filling	Mashed potatoes (powder reconstituted with water)
Cocoa powder reconstituted with water		Mashed potatoes (made from cooked potatoes)
Curd cheese		Reconstituted skim milk powder
Curd cheese with vanilla flavour		Milk drink with nut flavour
Whey drink peach flavour		Rice pudding (natural)
Whey drink cherry/banana flavour		Rice pudding (strawberry flavour)
Nougat creme		Rice pudding (chocolate flavour)
Sauce carbonara		Rice pudding (vanilla flavour)
Chocolate bar with milk/caramel filling		Rice pudding (cinnamon flavour)
Soy bean sprouts		Boiled whole grain rice
Quark		Scrambled egg
Herbal salt (10% in water)		Soy milk
		Soy milk-based dessert with caramel flavour
		Soy milk-based dessert with vanilla flavour
		Reconstituted whole milk powder
		Mousse au vanilla
		Pudding with vanilla flavour
		Vanilla sauce
