# Supplementary material for: Emetic Bacillus cereus Are More Volatile Than Thought: Recent Foodborne Outbreaks and Prevalence Studies in Bavaria (2007–2013)
Source: Biomed Res Int. 2014 May 8;2014:465603. doi: 10.1155/2014/465603 (PMC4033357; doi:10.1155/2014/465603)
Supplement: Supplementary file 1 — Figures S1, S2, S3: Examples of foods categorized being at low risk (Si), at risk (S2) or at high risk (S3) to promote cereulide formation in the presence of emetic B. cereus strains. The bioassay-based risk categorization was performed by inoculating 70 retail products and food ingredients with a lux based B. cereus cereulide reporter strain. The luminescence intensity, which corresponds to the amount of synthesized cereulide [24], was quantified in situ after incubation of the samples for 24 hours at 24°C (for details, see Material and Methods). Food samples were grouped into three main classes regarding their toxin formation capability via a software-assisted region-of-interest (ROI) analysis. The bioassay revealed that 44% of the foods could be categorized as high-risk foods, while the remaining 20% and 36% were categorized as risk or low-risk foods, respectively. Derived mean ROI values of each risk category and the corresponding determined threshold values are listed in Table S1. [file 465603.f1.pdf]

Supporting information

Figures S1, S2, S3: Examples of foods categorized being at low risk (S1), at risk (S2) and at high risk (S3) to promote cereulide formation in the presence of emetic *B. cereus* strains.

Fig: S1: low risk foods

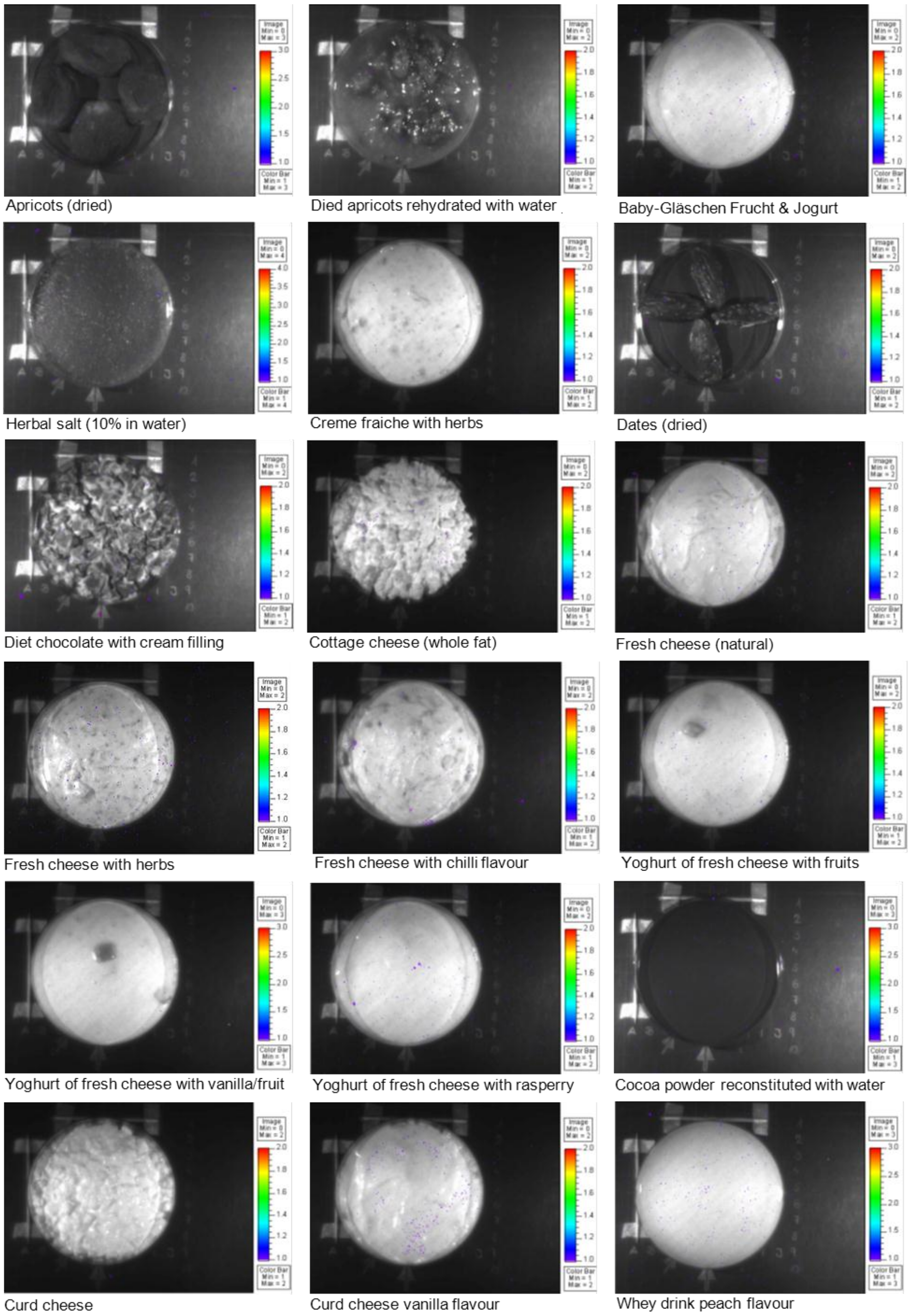

Fig: S2: risk foods

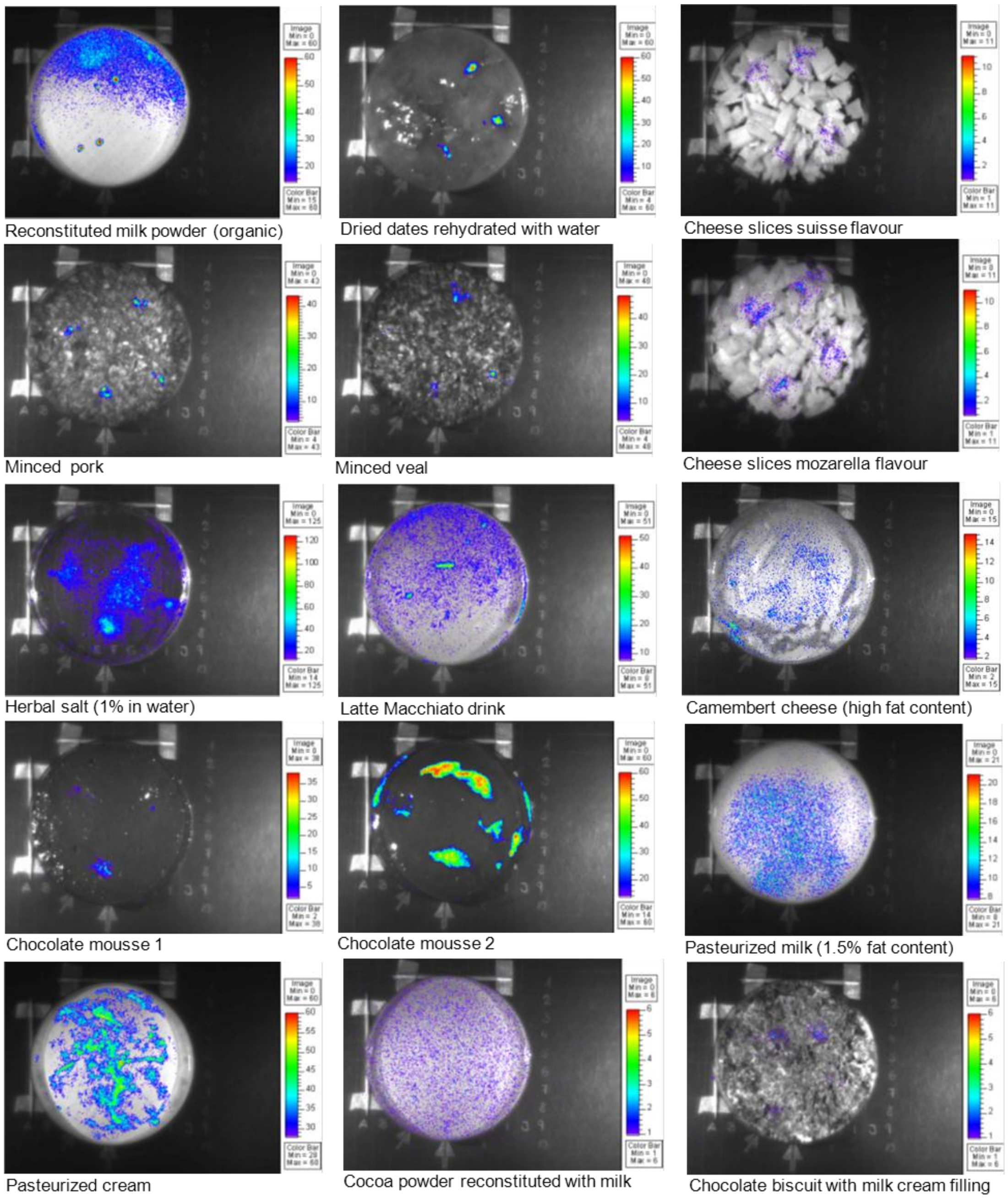

Fig: S3: high risk foods

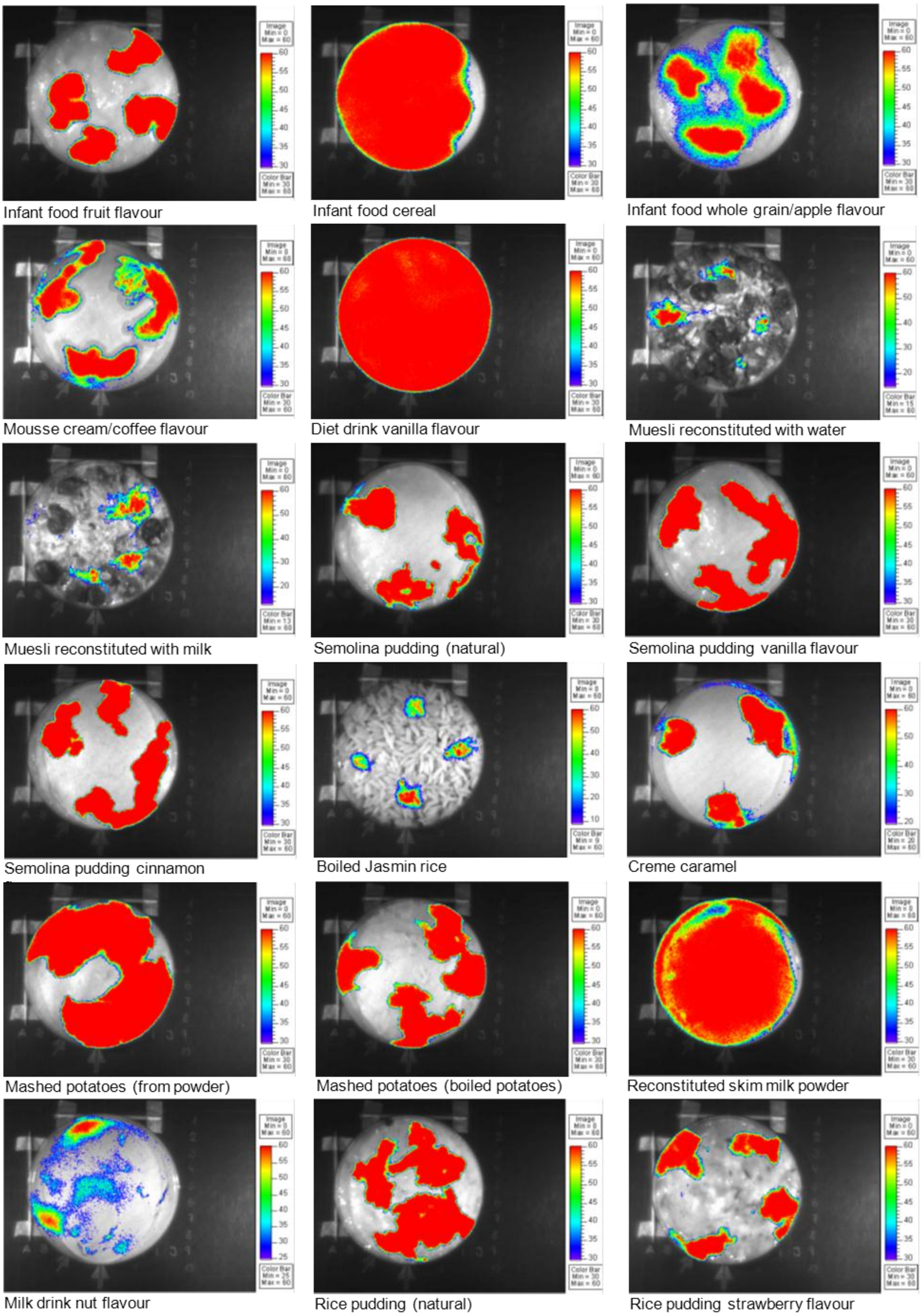

Table S1. Threshold values for software-assisted categorization of food products regarding their potential to support cereulide synthesis[a]

Food category	Mean ROI sum	Minimal ROI sum	Maximal ROI sum
Low risk	5.2E+02	4.2E+01	8.3E+02
Risk	2.9E+04	1.3E+03	6.7E+04
High risk	3.8E+06	2.1E+05	9.7E+06

[a] A set of 70 retail foods was inoculated with a *lux*-based *B. cereus* reporter strain for monitoring cereulide synthesis. Bioluminescence imaging was carried out with an ICCD camera system. The total photon count was quantified *via* region of interest (ROI) analysis as described in Material and Methods.
